# The relationship between deprivation, tumour stage and the systemic inflammatory response in patients with primary operable breast cancer

**DOI:** 10.1038/sj.bjc.6602096

**Published:** 2004-08-10

**Authors:** A M Al Murri, J C Doughty, A Lannigan, C Wilson, C S McArdle, D C McMillan

**Affiliations:** 1 University Department of Surgery, Glasgow Royal Infirmary, Glasgow G31 2ER, UK; 2 University Department of Surgery, Western Infirmary, Glasgow, UK; 3 Department of Surgery, Wishaw General Hospital, Lanarkshire, UK

**Keywords:** deprivation, systemic inflammatory response, C-reactive protein, breast cancer

## Abstract

The extent of deprivation (Carstairs deprivation index) was directly associated with the magnitude of the systemic inflammatory response (reduced albumin and elevated C-reactive protein, *P*<0.01) in patients with primary operable breast cancer (*n*=314). Deprivation was not associated with age, tumour size, tumour type, grade, and the proportion of patients with involved lymph nodes and oestrogen receptor status.

Breast cancer is the commonest female malignancy in the Western World. In the UK, the lifetime risk is now one in nine and the incidence is rising by about 2% per year (www.cancerresearchuk.org/stati stics).

It is now recognised that socioeconomic deprivation is associated with poor outcome in women with breast cancer (Schrijvers *et al*, 1995; McLaren and Bain, 1998; Thomson *et al*, 2001). For example, Thomson *et al* (2001), in a cohort of almost 22 000 women with primary breast cancer, reported a 10% survival difference between affluent and deprived. The basis of this relationship remains unclear. This difference does not appear to be related to tumour size or nodal status, nor access to hospital care, surgical management or the use of adjuvant therapy (Macleod *et al*, 2000; Brewster *et al*, 2001; Thomson *et al*, 2001). However, there is evidence to suggest that there is an association between deprivation and oestrogen receptor (ER) status, the deprived having more ER negative tumours (Thomson *et al*, 2001). However, ER status accounted for less than 20% of the difference in survival.

Recently, the systemic inflammatory response as evidenced by C-reactive protein, the prototypical marker of the systemic inflammatory response (Gabay and Kushner, 1999), has been shown to be associated with poor outcome in patients with colorectal and lung cancer (Scott *et al*, 2002; McMillan *et al*, 2003a). Furthermore, in patients with colorectal cancer, the presence of a systemic inflammatory response appeared to account for the effect of deprivation on cancer survival (McMillan *et al*, 2003b). To our knowledge, the nature of the relationship between deprivation, stage at presentation and the systemic inflammatory response in patients with primary operable breast cancer has not been previously examined.

The aim of the present study was to examine the relationship between deprivation, stage at presentation and the systemic inflammatory response in patients with primary operable breast cancer.

## PATIENTS AND METHODS

In total, 314 patients presenting with invasive primary operable breast cancer to three consultant surgeons (JCD, AL and CW) in two hospitals (Western Infirmary, Glasgow and Wishaw General Hospital, Lanarkshire) in the West of Scotland between July 2001–July 2003 were included in the study.

Prior to surgery, a blood sample was obtained for the measurement of albumin and C-reactive protein. At this time no patients showed clinical evidence of infection or other inflammatory conditions.

Age, deprivation category, tumour size, histological type and grade, oestrogen receptor status and lymph node status were recorded. The extent of deprivation was defined using the Carstairs deprivation index (Carstairs and Morris, 1991). This is an area-based measure derived from the 1991 census, using the postcode of residence at diagnosis, which divides the score into a seven-point index. For illustrative purposes, the results are presented by amalgamating the seven categories into three groups: affluent (categories 1 and 2), intermediate (categories 3–5) and deprived (categories 6 and 7). The Carstairs deprivation index has been extensively utilised in cancer patients (Edwards and Jones, 1999) and is particularly appropriate for use in the central belt of Scotland (MacKie and Hole, 1996).

The study was approved by the local Research Ethics committees.

*Blood parameters*: Routine laboratory measurements of C-reactive protein and albumin concentrations were carried out. The coefficient of variation was less than 5% as established by routine quality control procedures. The limit of detection of the assay is a C-reactive protein concentration of less than 6 mg l^−1^.

### Statistics

As appropriate, comparisons between patient groups were carried out using the *χ*^2^-test. Statistical analysis was based on the seven individual deprivation categories. In the absence of a predefined and recognised cutoff for circulating concentrations of albumin in patients with primary operable breast cancer, the data were analysed on the basis of tertiles. C-reactive protein concentrations were analysed on the basis of a two-point scale depending on whether C-reactive protein was undetectable or detectable (⩽6/>6 mg l^−1^). The correlation between C-reactive protein and albumin concentrations was performed using the Spearman's Rank correlation. Analysis was performed using SPSS software (SPSS Inc., Chicago, IL, USA).

## RESULTS

The clinico-pathological characteristics of patients with a diagnosis of invasive primary operable breast cancer (*n*=314) according to deprivation category are shown in Table 1

**Table 1:** Clinical and pathological characteristics of patients with invasive primary operable breast cancer according to deprivation

	**Deprivation (1–2)**	**Deprivation (3–5)**	**Deprivation (6–7)**	
	**(*n*=43)**	**(*n*=186)**	**(*n*=85)**	***P*-value**
Age (⩽50/>50 years)	11/32	46/140	13/72	
(%)	26/74	25/75	15/85	0.736
Size (⩽2/>2 cm)	24/19	160/72	50/35	
(%)	56/44	60/40	59/41	0.835
Ductal/lobular/other	36/4/3	153/17/16	67/12/6	
(%)	84/9/7	82/9/9	79/14/7	0.632
Grade (I/II/III)	5/17/20	40/87/59	13/49/22	
(%)	12/41/47	21/47/32	16/58/26	0.058
Involved lymph node (0/1–3/>3)	24/11/6	107/51/22	39/32/14	
(%)	58/27/15	60/28/12	46/37/17	0.726
ER (negative/positive)	7/36	45/140	18/66	
(%)	16/84	24/76	21/79	0.592
Albumin concentration (tertiles)	7/20/16	71/60/55	27/25/33	
(%)	16/47/37	38/32/30	32/29/39	<0.001
C-reactive protein (⩽6/>6 mg l^−1^)	36/7	124/62	47/38	
(%)	84/16	67/33	55/45	0.006

a Individual deprivation categories were used in the statistical analysis.

. In total, 14% of the patients were affluent and 27% were deprived. The median value of albumin in the lowest and highest tertiles was 40 and 46 g l^−1^ respectively. The median value of C-reactive protein in those patients with a detectable concentration was 9 mg l^−1^.

There was no significant relationship between the extent of deprivation, age, tumour size, tumour type, grade, and the proportion of patients with involved lymph nodes and ER status. In contrast, deprivation was associated with lower albumin (*P*<0.001) and higher C-reactive protein (*P*<0.01) concentrations.

Albumin concentrations were not related to age, size, type, grade, lymph node status or ER status. C-reactive protein was associated with increasing age (*P*=0.016) and ER negative tumours (*P*=0.035) but not with size, type, grade or lymph node status.

## DISCUSSION

In the present study, we examined a cohort of patients with primary operable breast cancer. Approximately a quarter of the patients were deprived. This is not surprising since half of the most deprived areas in the UK are in the West of Scotland (Gordon *et al*, 1999).

There was no significant relationship between the extent of deprivation and age, tumour size, type and grade and the proportion of involved lymph nodes. These results are consistent with previous studies (Brewster *et al*, 2001; Thomson *et al*, 2001). However, the relationship between deprivation and ER status shown by Thomson *et al* (2001) was not demonstrated in the present study, probably because our numbers were relatively small.

In the present study, deprivation was associated with lower albumin and higher C-reactive protein concentrations. These results are consistent with our previous study in patients with colorectal cancer, which demonstrated a significant association between deprivation, an elevated C-reactive protein and decreased survival (McMillan *et al*, 2003b). This relationship between deprivation, C-reactive protein and survival remains to be established in patients with primary operable breast cancer.

The factors underlying the relationship between the extent of deprivation and the systemic inflammatory response are not clear. However, obesity and smoking, which are associated with deprivation, are also known to be associated with elevated C-reactive protein concentrations (Tracy *et al*, 1997; Koenig *et al*, 1999). Furthermore, in colorectal cancer at least, there is increasing evidence that the presence of an elevated C-reactive protein concentration is associated with reduced survival (Nielsen *et al*, 2000; McMillan *et al*, 2003a).

In summary, the results of the present study demonstrated an association between the extent of deprivation and the magnitude of the systemic inflammatory response in patients with primary operable breast cancer.
